# Bilateral Renal Artery Thromboembolism During Thrombolytic Therapy for Embolism-Induced Acute Limb Ischemia

**DOI:** 10.1016/j.jaccas.2022.07.015

**Published:** 2022-10-05

**Authors:** Tuyen Van Le, Thai Truong, Hung Phung, An Ngo, Vien Thanh Truong, Duc Huu Nguyen

**Affiliations:** a Quang Tri General Hospital, Dong Ha, Viet Nam; b University of Medicine and Pharmacy at Ho Chi Minh City, Ho Chi Minh City, Viet Nam; c University of Pittsburgh Medical Center-McKeesport, McKeesport, Pennsylvania, USA; d Department of Internal Medicine, Nazareth Hospital, Philadelphia, Pennsylvania, USA

**Keywords:** catheter embolectomy, renal infarction, thrombus, CT, computed tomography, LAD, left anterior descending, LV, left ventricular, LVEF, left ventricular ejection function, TTE, transthoracic echocardiogram

## Abstract

We report a case of bilateral renal infarction following thrombolytic and anticoagulant therapy for left ventricular embolism–induced lower leg artery ischemia. Imaging demonstrated thrombi from the left ventricle leading to bilateral renal arterial occlusion. Catheter embolectomy and long-term oral anticoagulant therapy were initiated, and the patient recovered with no residual complications. (**Level of Difficulty: Intermediate.**)

## Central Illustration

**Figure:**
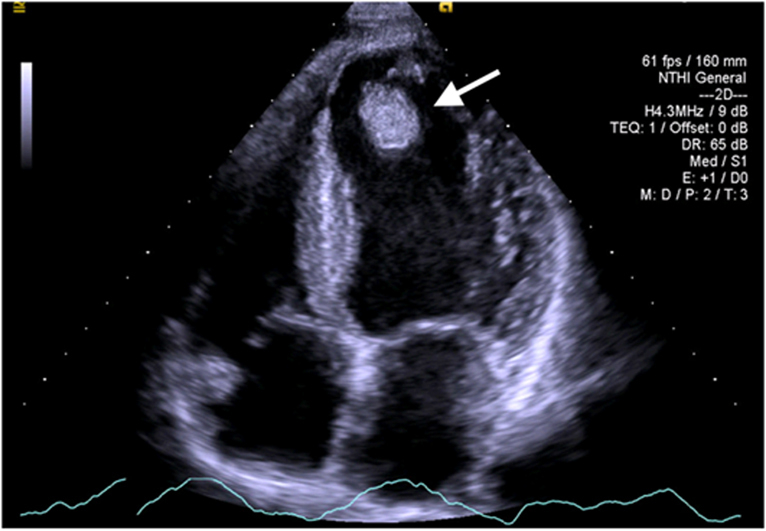

## History of Presentation

**Figure 1:**
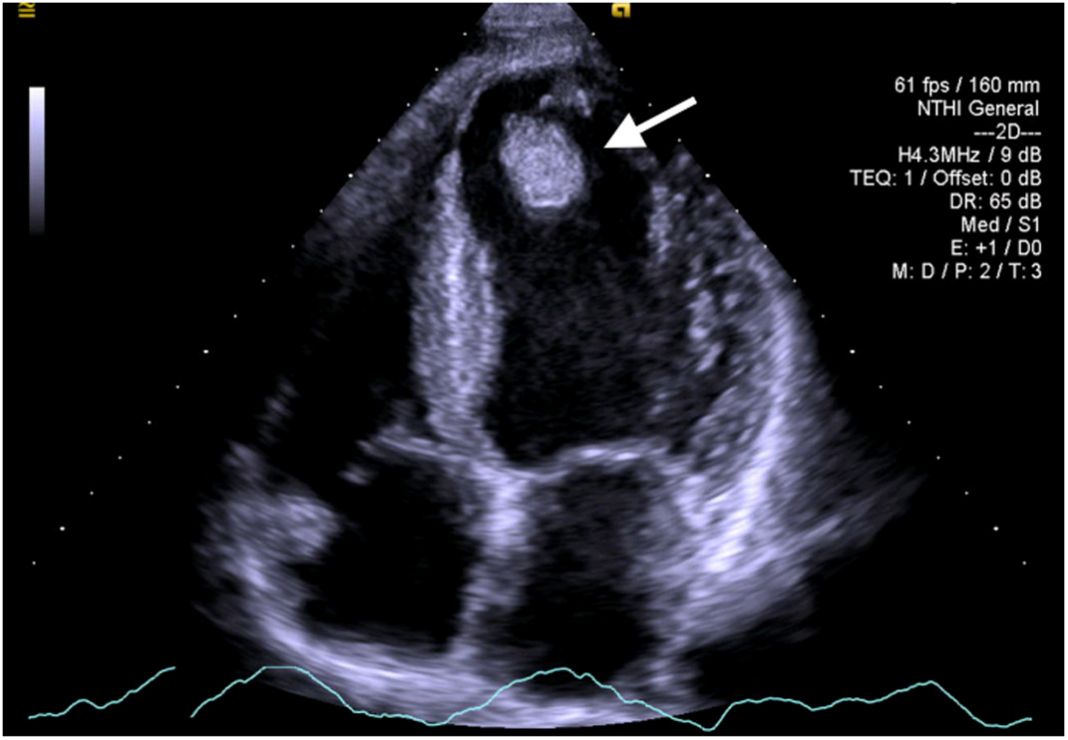
Transthoracic Echocardiogram on Admission A mobile thrombus measuring 21 × 15 mm is visible in the left ventricular apex **(arrow).**

A 59-year-old man with a history of heavy smoking was admitted to the emergency department because of 5 hours of left lower leg pain. His heart sounds were normal, and the lungs were clear. His left lower leg was cold and pale, with a weak left femoral artery pulse. No pulse was palpated at the left popliteal, dorsalis pedis, and posterior tibial arteries. His neurologic function was intact in both lower extremities. The arterial duplex sonogram revealed thrombi occluding the proximal left common femoral artery and 3 arteries below the left knee at the rate of 80% and 100% respectively. The electrocardiogram revealed sinus tachycardia with no definite ST-T-wave changes. The transthoracic echocardiogram (TTE) demonstrated significant left ventricular (LV) systolic dysfunction (left ventricular ejection function [LVEF], 30%-35%), apical akinesia, and a mobile thrombus measuring 21 × 15 mm in the LV apex (Figure 1, Video 1).

**Learning Objectives**

• To recognize that a new systemic embolic event may happen during the treatment of LV thrombus–induced embolism.
• To consider the approach of catheter embolectomy for new embolic events in the presence of major bleeding risk resulting from thrombolytic therapy

**Figure 2:**
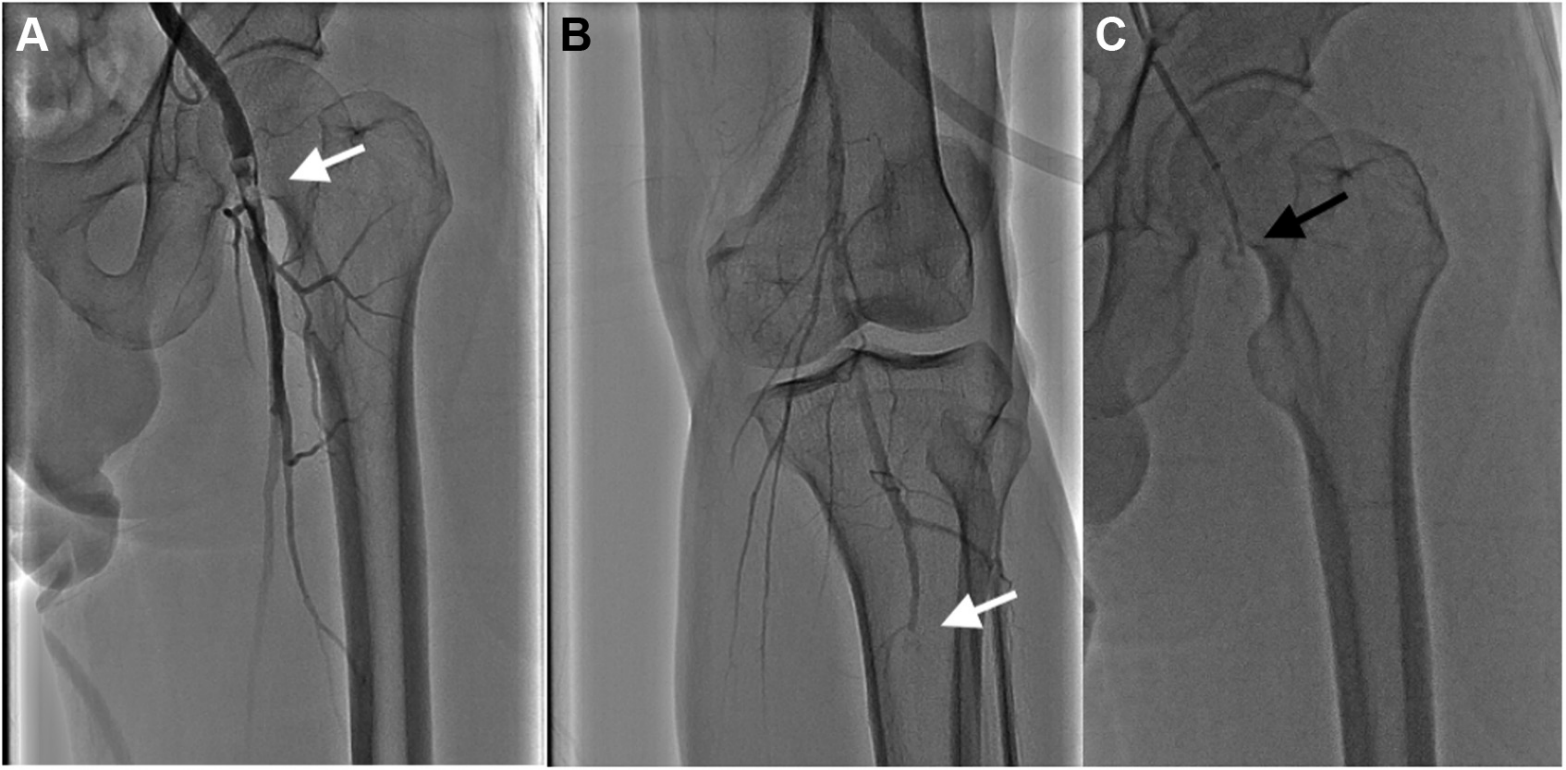
Catheter-Directed Thrombolysis Thrombi of the **(A)** left common femoral artery and **(B)** 3 arteries below the left knee **(white arrows). (C)** A multiple side-hole 5-F multipurpose catheter was advanced into the left common femoral artery under the guidance of a 6-F long sheath from the right femoral artery **(black arrow).**

The patient received a diagnosis of embolism-induced acute left lower leg ischemia. An intravenous injection of unfractionated heparin (100 U/kg) was immediately administered, followed by catheter-directed thrombolysis. A multiple side-hole 5-F multipurpose catheter (Multi Purpose MPA 1 SH, Cordis) was advanced into the left common femoral artery (Figures 2A to 2C) under the guidance of a 6-F long sheath from the right femoral artery. Recombinant tissue-type plasminogen activator and unfractionated heparin were concurrently infused through the catheter (1 mg/h) and sheath (200-500 U/h), respectively. An improvement in left lower leg ischemia was apparent 12 hours following the initiation of therapy; however, the patient began to have a new abrupt onset of bilateral, nonradiating, sharp, and constant flank pain without hematuria. The vital signs showed a body temperature of 36.3 ^°^C, arterial blood pressure of 140/90 mm Hg, heart rate of 75 beats/min, respiratory rate of 18 breaths/min, and saturated pulse oximetry of 98% on room air. No sign of guarding, rebound, or hematoma was found during the physical examination.

## Past Medical History

The past medical history included hypertension, ischemic heart disease, hypercholesterolemia, and no peripheral vascular disease.

## Differential Diagnosis

The differential diagnoses included psoas hemorrhage and renal artery embolism.

## Investigations

**Figure 3:**
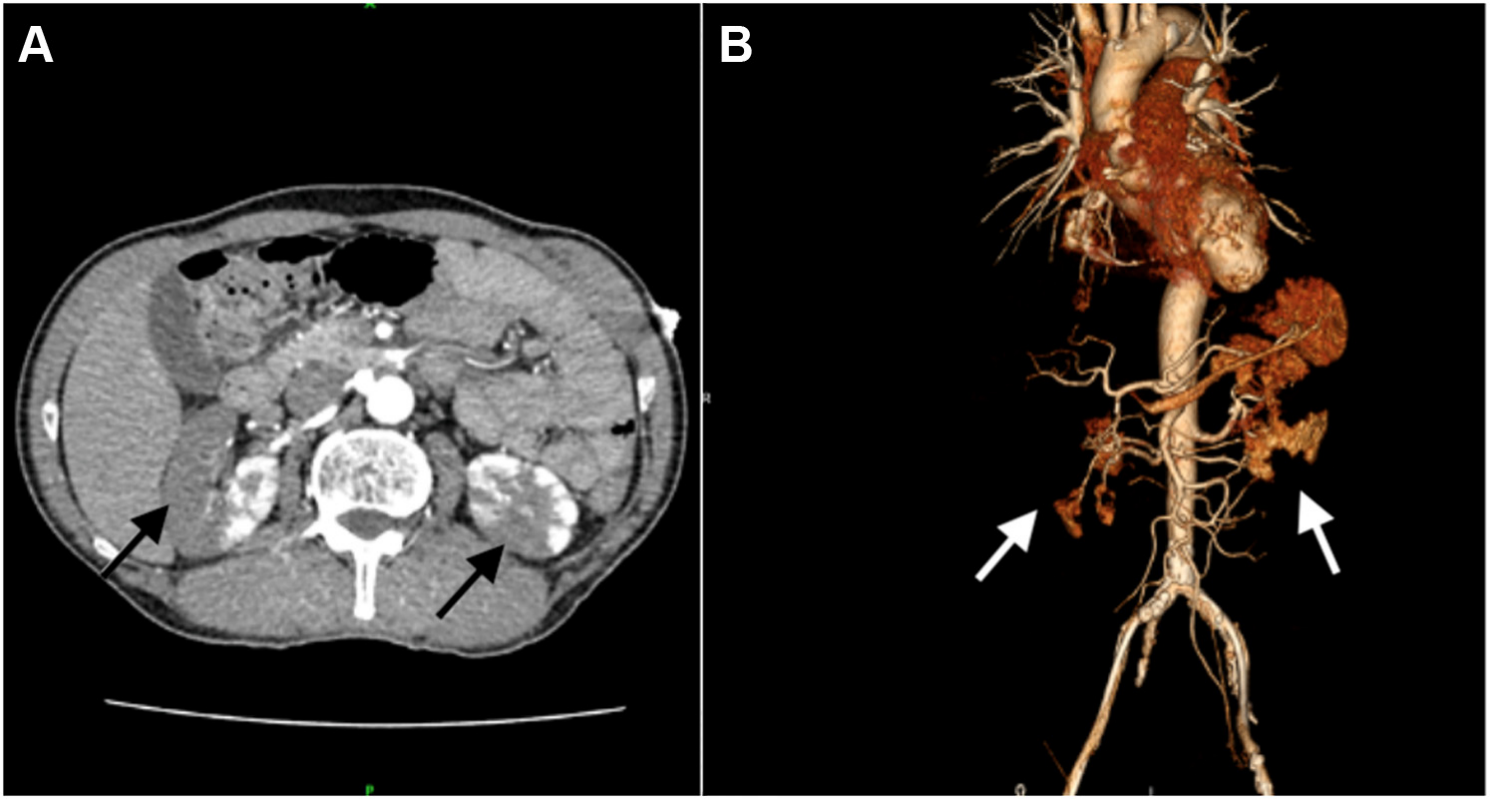
Renal Infarcts **(A)** Abdominal computed tomography scan with contrast enhancement revealed multiple extensive patchy infarcts in both kidneys **(black arrows). (B)** 3-dimensional reconstruction with barely visible of both renal tissue **(white arrows).**

**Figure 4:**
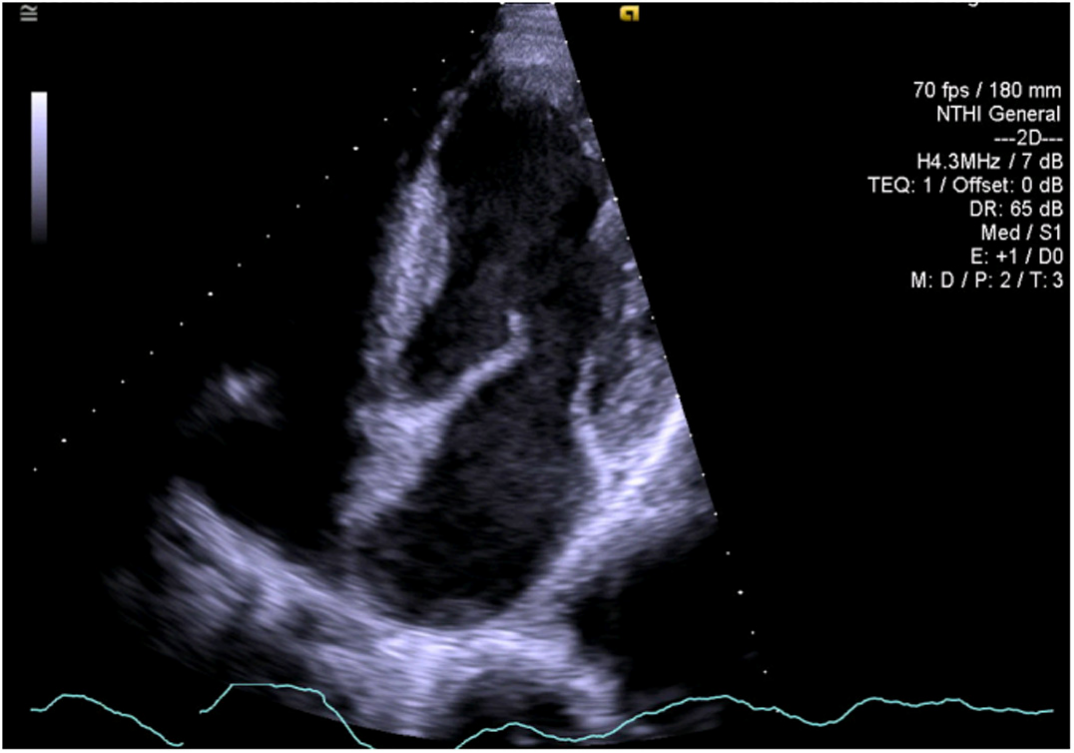
Left Ventricular Thrombus Resolution The transthoracic echocardiogram revealed complete resolution of the apical left ventricular thrombus.

**Figure 5:**
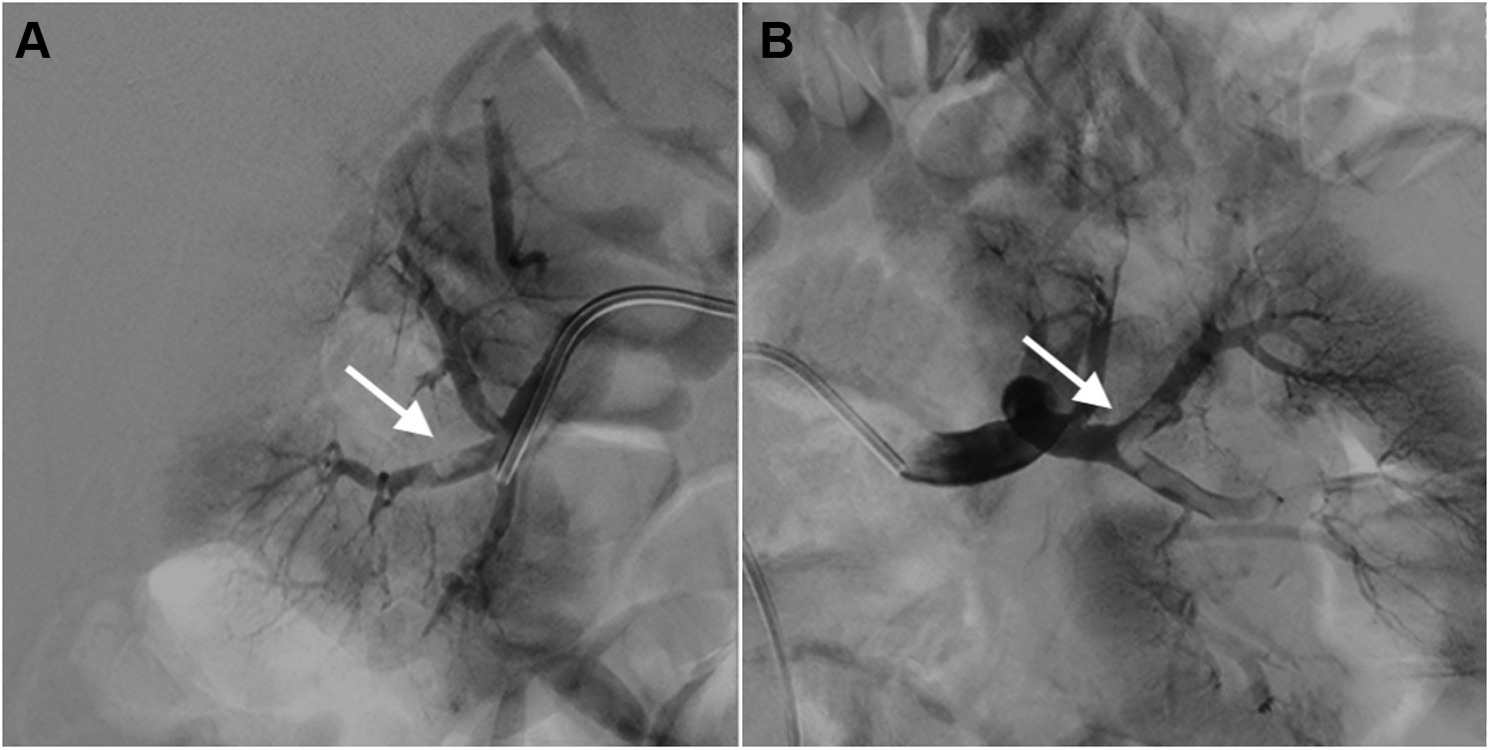
Renal Arterial Thrombi **(A and B)** A renal angiogram revealed extensive bilateral renal arterial thrombi **(arrows).**

An abdominal computed tomography (CT) scan with contrast enhancement revealed multiple extensive, patchy infarcts in both kidneys (Figures 3A and 3B). At the same time, serial TTE showed complete resolution of the apical LV thrombus (Figure 4, Video 2). A renal angiogram detected extensive bilateral renal arterial thrombi (Figures 5A and 5B). The patient’s serum creatinine level increased from 97 to 165 μmol/L. The hematologic and coagulation test results were unremarkable.

## Management

**Figure 6:**
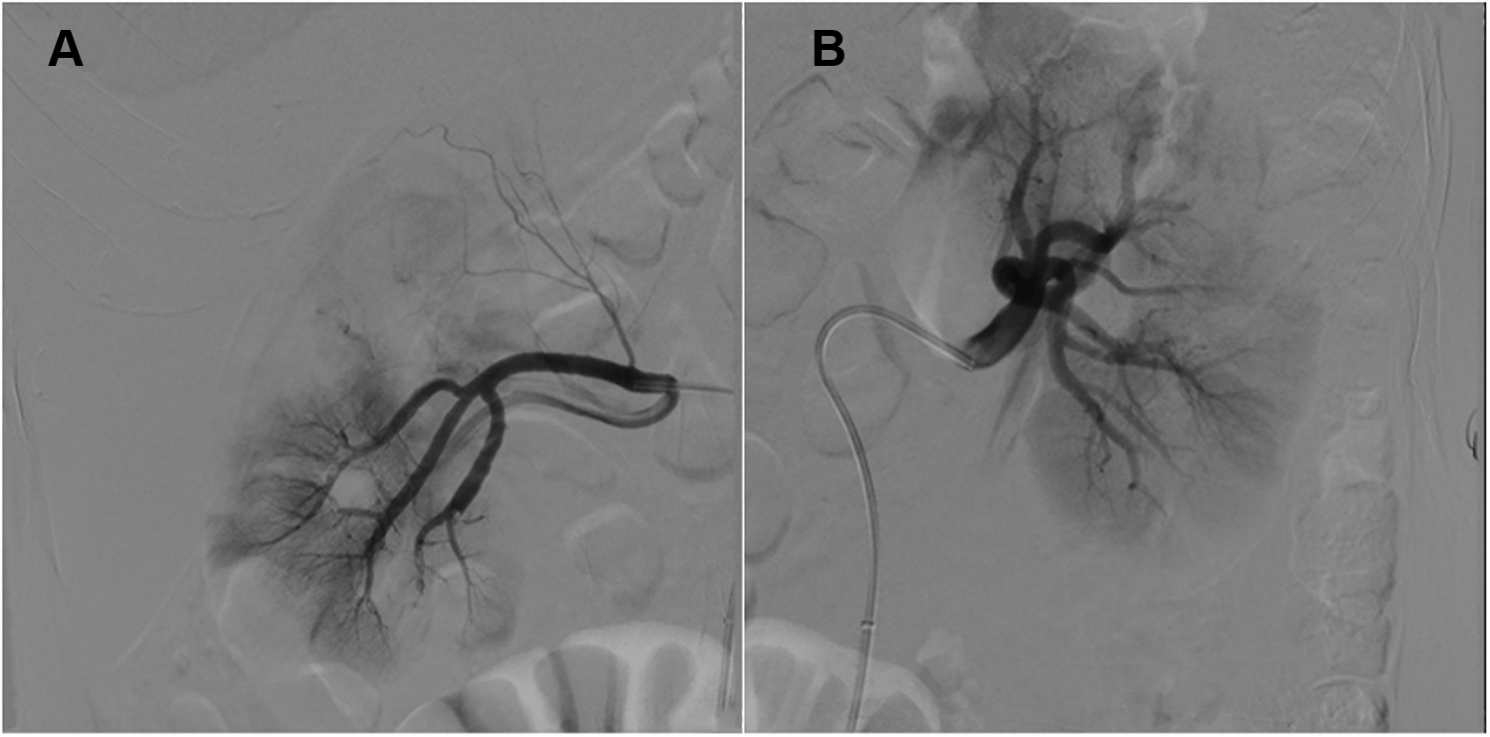
Renal Perfusion Restoration **(A and B)** Postinterventional images show restoration of renal perfusion with excellent kidney blush.

**Figure 7:**
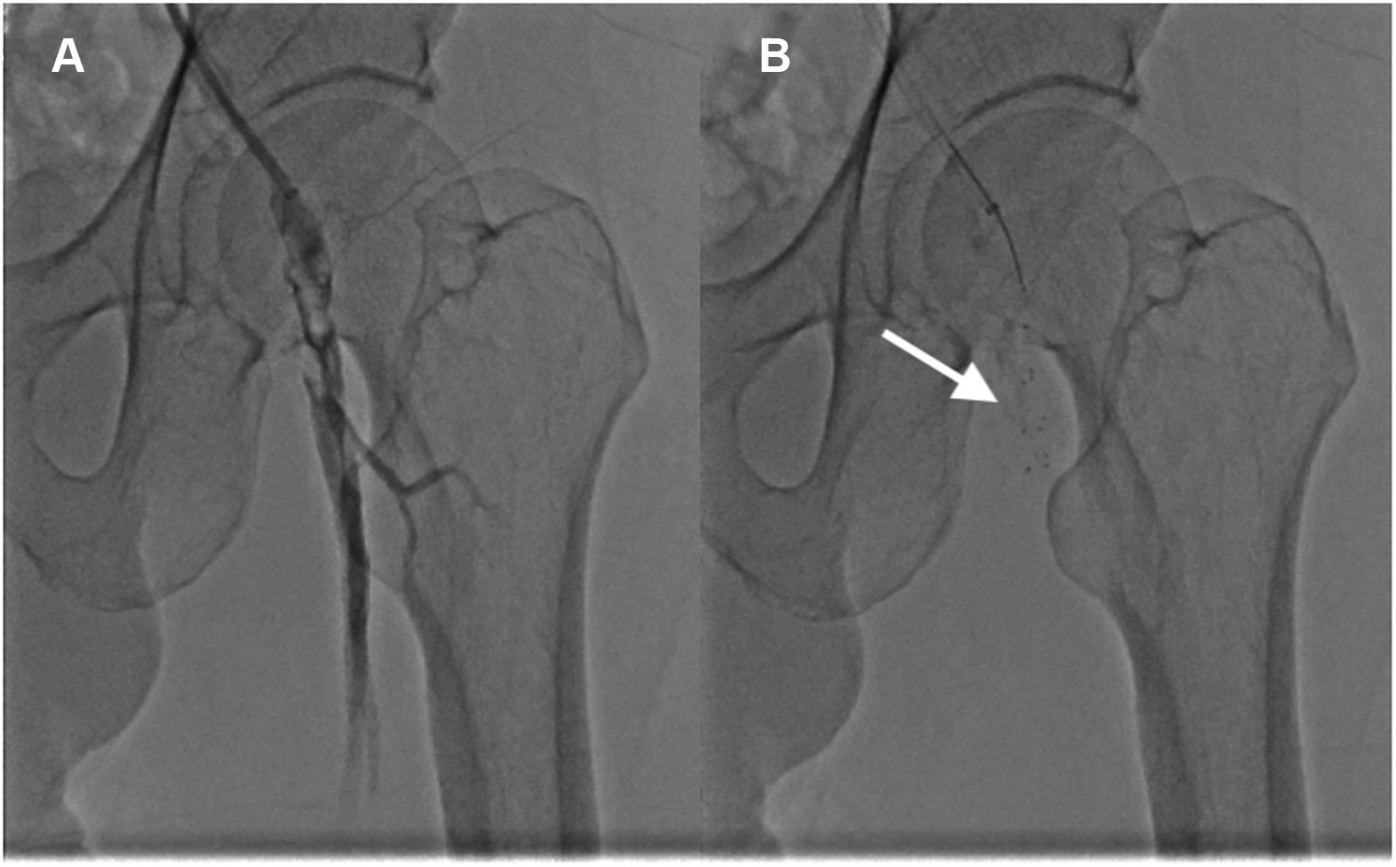
Common Femoral Artery Thrombus Removal **(A and B)** Thrombus removal. **(B)** A 3MAX (Penumbra) reperfusion catheter combined with a Solitaire (ev3) revascularization device **(arrow)** retrieves a large amount of **(A)** thrombi from the common femoral artery.

**Figure 8:**
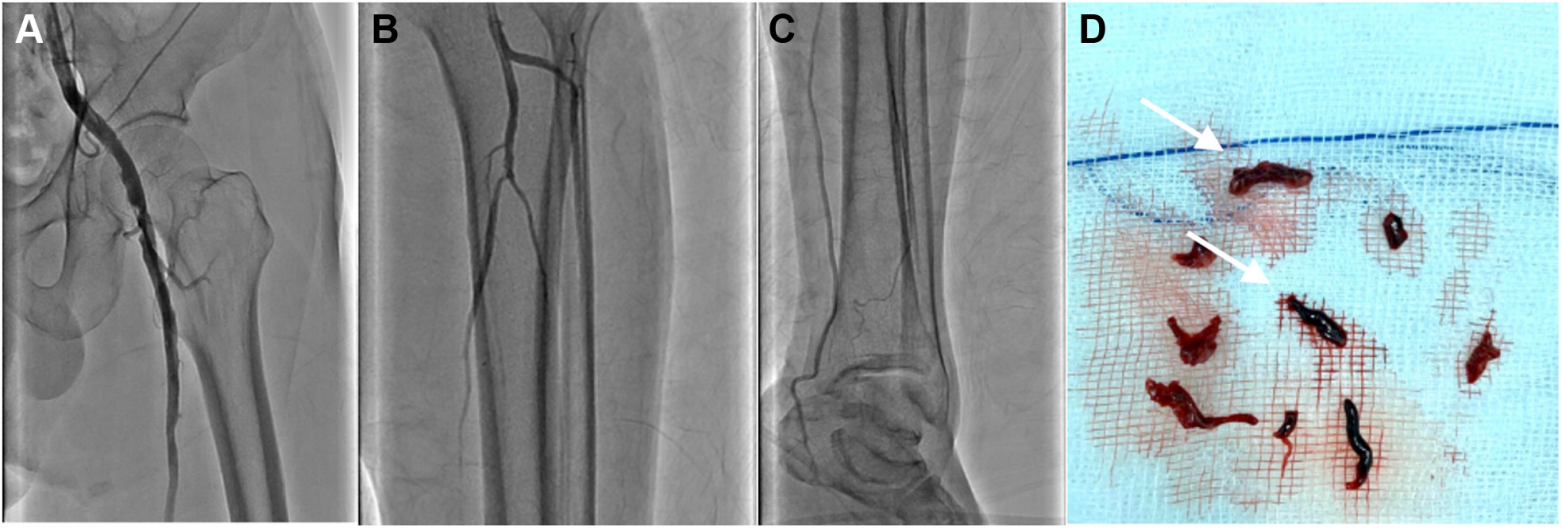
Circulation Restoration and Retrieved Thrombi Angiography after successful recanalization at **(A)** the common femoral artery and **(B and C)** the arteries below the knee. **(D)** Thrombi from the renal and femoral arteries **(arrows).**

Embolism-induced acute bilateral renal infarction was suspected. The bilateral renal arteries were cannulated by a 6-F long sheath (Epsylar, Optimed) with a Yashiro-tip catheter (Terumo). A 3MAX reperfusion catheter (Penumbra) was then advanced over a hydrophilic 0.014-inch guidewire (Whisper Extra Support, Abbott Vascular) and combined with a Solitaire revascularization device (ev3) to retrieve a large amount of thrombus from the bilateral segmental arteries. Postinterventional images found restoration of renal perfusion with excellent kidney blush (Figures 6A and 6B). The procedure was also performed to recover the circulation of the left lower extremity fully (Figures 7A and 7B and 8A to 8D).

**Figure 9:**
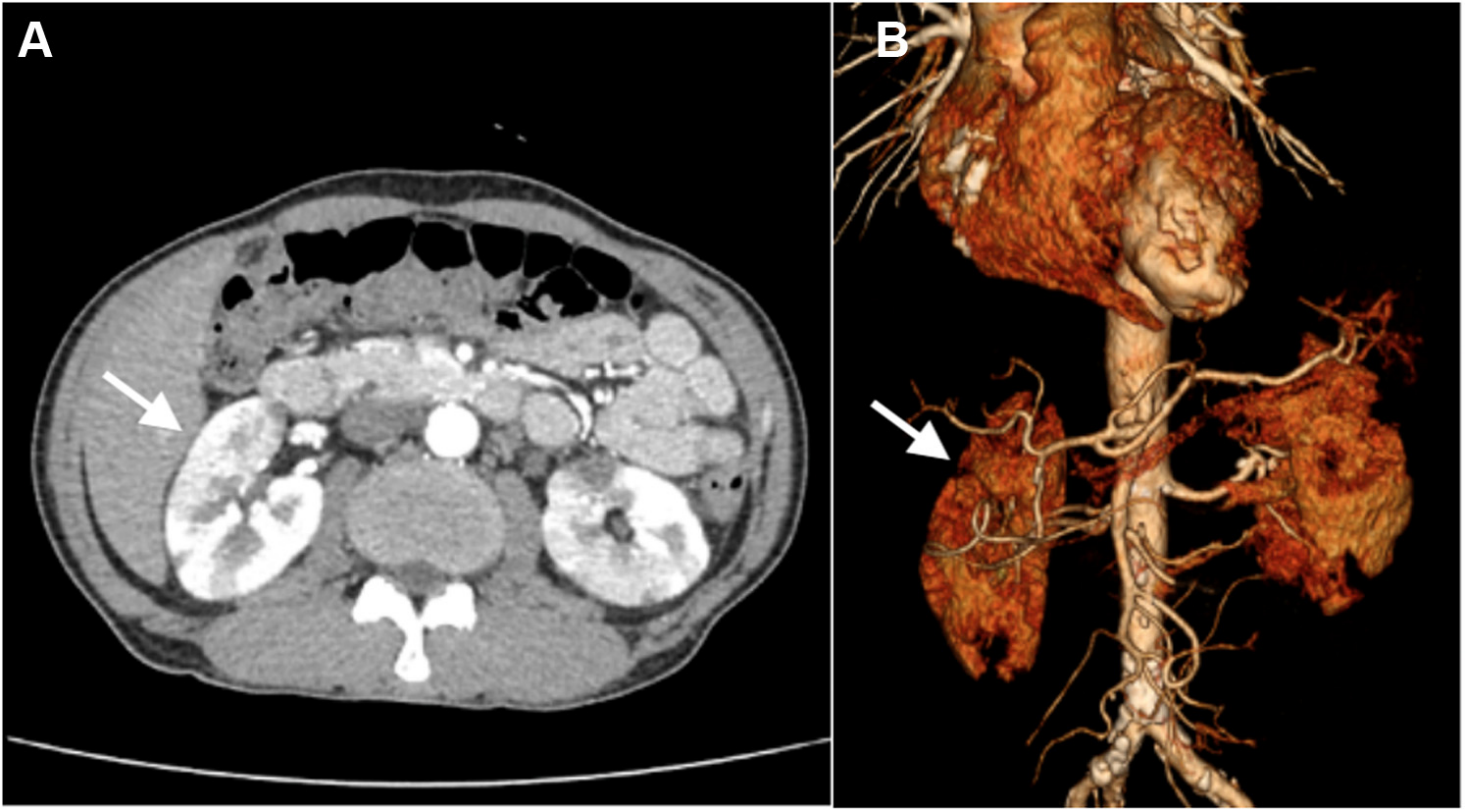
Renal Thrombi Resolution **(A and B)** Computed tomography demonstratedcomplete resolution of the renal thrombi **(arrows).**

The patient completely recovered, with no additional complications following the foregoing interventions. One week later, his serum creatinine level returned to normal (74 μmol/L), and a CT scan demonstrated complete resolution of the renal thrombi (Figures 9A and 9B). The patient also underwent primary invasive coronary angiography, which revealed atherosclerotic stenosis involvement of the left anterior descending (LAD) artery, the left circumflex artery, and the right coronary artery at a rate of 95%, 70%, and 80% respectively. Reperfusion was performed in the LAD artery by using percutaneous coronary intervention deploying 2 stents.

## Follow-Up

He was discharged on day 10 with a regimen of oral warfarin (1 mg/day), clopidogrel (75 mg/day), aspirin (81 mg/day), atorvastatin (40 mg/day), lisinopril (5 mg/day), and bisoprolol (5 mg/day) at home. Aspirin was withdrawn after 6 months, and warfarin was replaced indefinitely with a new oral anticoagulant agent after 12 months. The patient remained stable during the follow-up.

## Discussion

LV thrombosis is often associated with acute myocardial infarction. The triad of Virchow, which consists of hypercoagulation, endothelial cell injury, and blood stasis, following an acute myocardial infarction can precipitate LV thrombosis.^1^ Although our patient had no past medical history of myocardial infarction, an LV thrombus most likely developed as a result of the blood stasis in the presence of apical akinesia and a significantly reduced LVEF, as well as damaged subendocardial tissues from prolonged myocardial ischemia.

The systemic embolism secondary to LV thrombus is theoretically described yet uncommonly reported. Risk factors include severe congestive heart failure, diffuse LV dilation and systolic dysfunction, previous embolization, advanced age, and protruding or mobile thrombi.^1^ The finding of thrombi in our patient’s lower extremity arterial sonogram was consistent with embolism in light of: 1) no past medical history of peripheral vascular disease suggestive of atherosclerotic thrombosis of the extremity artery; and 2) mobile thrombus in the left ventricle with significant LV systolic dysfunction. His renal infarcts also most likely resulted from embolism because they coincided with the disappearance of LV thrombus. A similar case of bilateral renal and popliteal artery embolism was also reported but occurred concurrently following a myocardial infarction in a young woman with long-term oral contraceptive use.^2^

There is no current consensus on the management of LV thrombus. More evidence is required to confirm the utility of anticoagulant agents, given their debatable safety and efficacy.^3^^,^^4^ According to a retrospective study, although surgical interventions tended to be more effective than anticoagulant therapies in terms of thromboembolic prophylaxis, the difference was actually insignificant.^5^ Despite our patient’s LV thrombus, the priority was placed on preventing imminent extremity ischemic necrosis. This objective was, however, complicated by an embolism-induced renal infarction. Therefore, the event was probably secondary to the dislodgment of LV thrombus following thrombolysis.

Although anticoagulation with or without thrombolysis is the mainstay of treatment, there are currently no guidelines for the management of renal artery embolism. Catheter-directed thrombolysis has been previously described as a safe and efficient treatment strategy.^6^, ^7^, ^8^ Surgical revascularization is not favored because of its higher mortality rate.^7^^,^^9^ Given a higher risk of bleeding following thrombolytic and anticoagulant therapy, catheter embolectomy was selected to reperfuse the patient’s renal artery. To our knowledge, this is the first case report of successful catheter embolectomy in a patient presenting with concomitant risk factors for significant bleeding and renal artery embolism. European guidelines recommend a vitamin K antagonist for at least 3 to 6 months to prevent recurrent thrombosis following myocardial infarction, whereas U.S. guidelines advise using this agent indefinitely in patients with no increased risk of bleeding.^1^ As indicated by his HAS-BLED score of 2, our patient had a moderate risk for a major bleeding event and therefore could be administered a long-term anticoagulant agent cautiously.^10^

## Conclusions

Patients with severe coronary artery disease have an increased risk of LV thrombosis and therefore also have an increased risk of systemic embolism. Although anticoagulant agents are often used to resolve LV thrombus, final consensus for this approach is pending. The therapeutic strategy for LV thrombus–induced embolism should be proceeded with caution because of the possibility of new embolic and major bleeding events.

## Funding Support and Author Disclosures

The authors have reported that they have no relationships relevant to the contents of this paper to disclose.

## Appendix

Supplemental Video 1 Transthoracic Echocardiogram Demonstrating Significant Left Ventricular Systolic Dysfunction, Apical Akinesia, and a Mobile Thrombus Measuring 21 × 15 mm in the LV Apex

Supplemental Video 2 Transthoracic Echocardiogram Revealing Complete Resolution of the Apical LV Thrombus

## References

[1] Delewi R., Zijlstra F., Piek J.J. (2012). Left ventricular thrombus formation after acute myocardial infarction. Heart.

[2] Liao S.F., Lee C.H., Wu L.S., Li C.H., Chen H.Y. (2018). Left ventricular thrombus and systemic embolism after painless myocardial infarction in a young female. Hong Kong J Emerg Med.

[3] Massussi M., Scotti A., Lip G.Y.H., Proietti R. (2021). Left ventricular thrombosis: new perspectives on an old problem. Eur Heart J Cardiovasc Pharmacother.

[4] Lattuca B., Bouziri N., Kerneis M. (2020). Antithrombotic therapy for patients with left ventricular mural thrombus. J Am Coll Cardiol.

[5] Lee J.M., Park J.J., Jung H.W. (2013). Left ventricular thrombus and subsequent thromboembolism, comparison of anticoagulation, surgical removal, and antiplatelet agents. J Atheroscler Thromb.

[6] Wright M.P.J., Persad R.A., Cranston D.W. (2001). Renal artery occlusion. BJU Int.

[7] Kansal S., Feldman M., Cooksey S., Patel S. (2008). Renal artery embolism: a case report and review. J Gen Intern Med.

[8] Silverberg D., Menes T., Rimon U., Salomon O., Halak M. (2016). Acute renal artery occlusion: presentation, treatment, and outcome. J Vasc Surg.

[9] Moyer J.D., Rao C.N., Widrich W.C., Olsson C.A. (1973). Conservative management of renal artery embolus. J Urol.

[10] Camm A.J., Kirchhof P., Lip G.Y. (2010). Guidelines for the management of atrial fibrillation: the Task Force for the Management of Atrial Fibrillation of the European Society of Cardiology (ESC). Europace.

